# Smoking prevention intervention with school classes in university hospital by thoracic surgeon und pulmonologist. The Zurich prevention project

**DOI:** 10.1016/j.pmedr.2022.101964

**Published:** 2022-08-30

**Authors:** K. Furrer, M.M. Schuurmans, M. Hebeisen, S. Schulte, D. Schneiter, W. Weder, I. Opitz, S. Hillinger

**Affiliations:** a Department of Thoracic Surgery, University Hospital Zurich, Switzerland; b Division of Pulmonology, University Hospital Zurich, Switzerland; c Epidemiology, Biostatistics and Prevention Institute, University of Zurich, Switzerland

**Keywords:** Tobacco smoking prevention, Schoolchildren, Adolescent, Smoking cessation interventions, Smoking adverse effects, CI, Confidence interval, IQR, Interquartile range, i.e, Id est, OR, Odds radio, SD, Standard deviation, RCT, Randomized control trial, BASEC, Business Administration System for Ethics Committees

## Abstract

Smoking prevention in schoolchildren to inform and prevent smoking initiation has been widely studied; however, the potential effect of interventions provided in a hospital setting is unknown. An intervention program named “Schoolchildren smoking prevention in the hospital” was developed in which the health aspects of smoking and its individual consequences were presented in an interactive informational event provided by a thoracic surgeon and a pulmonologist. We aimed to assess the feasibility and the short-term effect of smoking-related knowledge improvement in schoolchildren in a hospital setting.

Scholars of 45 classes in Canton of Zurich in Switzerland filled in an anonymous 5-item questionnaire with questions on general knowledge about smoking. The answers were evaluated in this prospective observational cohort study. The primary endpoint was to compare the knowledge improvement by interpretation of answers before-and-after the smoking prevention intervention. Additionally, the performance of children was compared after setting up an overall score and specific subgroups according to gender and school-level.

Between Jan 2010, and Oct 2019, schoolchildren aged 10 to 16 years participated in this intervention program and completed the questionnaire before (N = 1270) and after (N = 1264) the intervention. The amount of correctly answered questions increased from 40% (±20) before to 81% (±17), p < 0·0001 after the educational session.

An intervention program on health effects of smoking provided by lung specialists in the hospital is feasible, well received, leads to a substantial increase of knowledge, and hopefully can be further explored in the development of smoking prevention programs for schoolchildren.

## Highlights

• Smoking prevention for scholars by lung specialists in hospital is feasible.
• The intervention program for pupils on health effects of smoking is well received.
• The interview with a lung transplant recipient and lung function testing are highly rated.
• Smoking prevention doubles smoking-related knowledge of scholars aged 10–16.

## 1 Introduction

Tobacco use kills one person every-four seconds and is globally responsible for 56·9 million deaths per year. Smoking cessation is the single most efficient way to avoid a substantial proportion of these tobacco-related deaths. An estimated one billion people will die of tobacco consumption associated diseases in the 21st century based on its two to three times increase in mortality of smokers compared to never smokers and leading to a shortened life expectancy by 10 years (Global, 2017, Jha and Peto, 2014, Pirie et al., 2013, Thun et al., 2013).

Currently 50% of young males and 10% of young females begin to smoke early (Jha and Peto, 2014). Worldwide, about half a billion of the smoking children and adults are younger than 35 years and only a small proportion of them will be able to quit smoking (Giovino et al., 2012). For children smoking initiation frequently starts with cigarette experimentation due to peer pressure and exposure in the school setting. In the meantime, other devices have appeared, such as electronic cigarettes and “heat not burn” products, which are often even more attractive to young people. The overall time point of smoking initiation is thought to be around age 9 and older, however this aspect is strongly dependent on the sociocultural background. A recently reported smoking rate of 16% in 11–15-year-old schoolchildren in UK, decreased from 49% in 1996 (Centre NI, 2018). In the first school years, scholars are receptive for experimentation and highly susceptible to the effects of nicotine from cigarettes leading rapidly to addictive behaviour.

The consequences of tobacco smoking in children and adolescents are potentially reversible, but there are only a few high-quality randomized controlled trials that have examined the benefits of prevention and treatment in primary healthcare settings (Thombs et al., 2017, Brinker et al., 2016). In addition, interventions that work for adults do not necessarily work well for schoolchildren and adolescents. Available evidence suggests that providing brief information and advice may help prevent smoking initiation among children and adolescents aged 5 to 18 years (Thombs et al., 2017).

In general, the best method to prevent the consequences of smoking is to reduce tobacco use and exposure to smoke. This is supported also by the WHO Framework Convention on Tobacco Control, which outlines global tobacco control and its health, social, environmental, and economic costs (Hoffman et al., 2019). Tobacco education as a part of the WHO efforts is particularly important for children and young adults.

The hospital environment as setting to influence scholars and young adolescents can be an advantage for tobacco education events since it leads to an authentic contact with patients and health professionals.

For the last 20 years different research groups have used five types of curricula in schools, each based on a different theoretical orientation including information-only, social competence, social influence, combined social competence with social influences curricula and multimodal approaches (Tobler et al., 2000, Thomas et al., 2015). Social competence interventions support adolescents to learn how to ignore offers to smoke by improving their personal and social interactions. Adolescents are informed about a combination of skills to improve problem solution, decision-making, self-control, self-esteem, assertiveness, and strategies to deal with stress, and to deal with general personal or media influences (Thomas et al., 2015). Social influence interventions focus on teaching adolescents to recognise social forces that promote substance use and to refuse tobacco offers, especially in pressure and high-risk situations that may directly or indirectly tempt an adolescent to smoke (Thomas et al., 2015). The combination of these two forms leads to the development of multimodal school curricula and tobacco prevention programs involving parents and community members (Thomas et al., 2015). There are some interventions for which there is convincing evidence, such as well-designed media campaigns with engaging, emotional, and vivid real-life stories. These can reduce tobacco use, increase quit attempts, reduce initiation rates, and reduce exposure to second-hand smoke (Brinker et al., 2016, Wrotgte, 2019, McAfee et al., 2013, Xiao et al., 2013, Bala et al., 2017, Lisboa et al., 2019).

We conducted a smoking prevention project, provided by thoracic surgeons and pulmonologists, to inform children about the health consequences of smoking in a hospital setting and to potentially influence the frequency of smoking uptake among these youths. The primary objective is to evaluate the feasibility and short-term effect on knowledge of a hospital-based intervention for schoolchildren. A second objective was to assess which type of provided knowledge topics are the most attractive for schoolchildren.

## 2 Material and methods

### 2.1 Description of intervention

Smoking prevention intervention sessions were carried out prospectively from January 2010 to October 2019 in 45 different classes. Most interventions lasted 2·5 h with 15 to 20 min break between the two components of the intervention program. Before and after the intervention, a survey was carried out and analyzed in the form of a before-and-after observational cohort study.

Briefly, classes with 20–30 children and adolescents were signed up by their teachers in direct contact with one of the investigators (SH or MS). The request for an educational session in which this intervention should fall was met in all cases.

The intervention was implemented on the campus of the University Hospital, so scholars are in contact with physicians and patients in their usual setting. The school classes are welcomed in the reserved lecture halls by both physicians and after a brief introduction, an age-adapted and well comprehensible knowledge questionnaire was filled in by the scholars (5 min). This anonymous questionnaire included school class designation, age, and gender of scholar. The same questionnaire was distributed at the end of the intervention.

The questionnaire contained the following questions and possible answers:

Question 1: What parts of the body (organs) are damaged by smoking?

Question 2: Do increased cigarette prices result in a reduction of smoking?

Question 3: Does smoking only 1–4 cigarettes per day over many years not carry significant health risks?

Question 4: Most smokers believe that they can quit smoking all by themselves. How many adults can quit smoking all by themselves?

Question 5: Is water pipe/hookah/shisha smoking not as dangerous as cigarette smoking?

A detailed description of the intervention and the school system in the Canton of Zurich are included in the supplementary material.

One of the key messages of this section was that it is much more difficult to quit smoking than to refrain from smoking as a young person. Subsequently, two video sequences of minimal invasive operations were shown, presenting the lungs of a non-smoker and, in the second sequence, the lungs of a heavy smoker with emphysema and lung cancer resected with the surgical instruments. These sequences were explained in detail by the thoracic surgeon and the subsequent questions of the schoolchildren concerning the slide show and the video were answered. At the conclusion of both parts of the intervention, we provided age-appropriate internet links with general information on the topic of addiction prevention.

We further emphasized that this intervention was intended to influence their lives and development and we asked the scholars to be tolerant with adults (their parents) who may be smokers and have similar difficulties to stop smoking as they have heard about during the current intervention session. This comment was necessary to prevent the young people from reacting strongly to adults smoking shortly after the sessions (as initially often reported by the teachers).

The questionnaires are completely anonymized and do not include personal information. The parents were informed by the teacher about the excursion to the hospital including the educative lesson and evaluation of the scholars knowledge. From the perspective of the ethics committee, an additional informed consent was not considered to be necessary and from an ethical point of view, there are no objections concerning the project (Ethics request number-2022–00035).

### 2.2 Statistical analysis

Answers to the knowledge questionnaire were compared before and after the educational session (control and intervention questionnaires, paired study design). Descriptive analysis in enclosed tables were shown for demographics, individual questions, and overall score of all questions (0–15 points), for the control and intervention questionnaire. Descriptive statistics include means and standard deviations (SD) for continuous and score variables, numbers, and percentages of total for categorical variables. All questions with unclear answers were counted as missing, irrespective of their cause (don’t know, inconclusive, no reply). From the correct answers, an overall score was calculated as follows: The sum of the number of items ticked in question 1 plus 3 for the correct answer to questions 2 and 5 plus 1 for the correct answer to questions 3 and 4 amounts to a maximum of 15 points. The higher attribution of points for certain questions was related to the importance of the questions for the target populations (youths). The answers to all questions before and after the intervention were compared by statistical tests.

Special versions of statistical tests were used because the pairing of the data was not recorded (supplementary material). Overall scores in different subgroups of scholars were compared by the standardized mean difference (SMD) and in the form of Hedge’s g with 95% CI. The bigger the difference, the higher the SMD. R version 4.0.3 (2020–10-10) was used for all statistical analyses (R Development Core Team, 2020) and all results were reported according to STROBE guidelines (von Elm et al., 2007).

## 3 Results

### 3.1 Cohort description and overall comparison

**Fig. 1:**
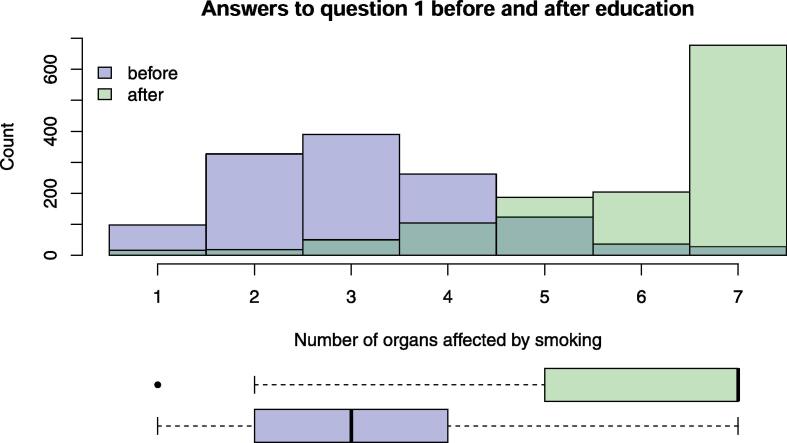
Number of selected question 1 items are plotted before and after the educational session as an overlay of two histograms and in boxplots. The correct answer is to select all 7 items. Total population N = 1270/1264.

**Fig. 2:**
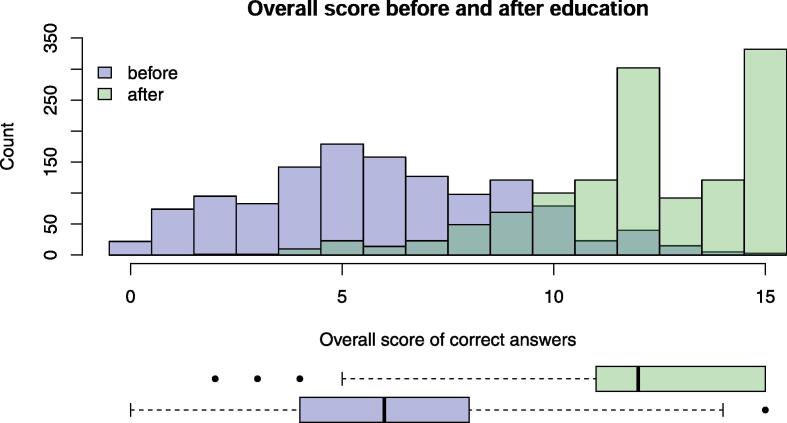
Overall score of correct answers is plotted before and after the educational session as an overlay of two histograms and in boxplots.

2534 five-item questionnaires were filled in twice by schoolchildren aged 10 to 16 years, and 1270 children participated in the intervention. About the same number of girls and boys participated in the educational sessions (Table 1) resulting in response rate of 99·3%. More than two thirds of the children were 13 or 14 years old and went to the seventh or eighth class of secondary school. Most secondary schoolchildren attended A or B school levels. The distribution of the intervention’s questionnaire items before- and after the educational session and corresponding results of statistical tests comparing answers before and after the educational event are presented in Table 2. The percentages of correctly answered questions increased significantly for all questionnaire items after the educational session (Table 2). In all questions, more than 50% of the students marked the correct answer after the intervention, in the last three questions more than 90% did so. The number of correct answers to question 1 increased from a median of 3 (Interquartile range (IQR) 2–4) to 7 (IQR 5–7) (Fig. 1), of 7 items. The overall score increased from a mean (±SD) of 6 (±3) points to 12 (±3) points where the maximum score was 15 points (Fig. 2).

**Table 1:** Distribution of schoolchildren characteristics before and after intervention with educational session, based on evaluation of the questionnaires. Percentage of missing data designates the missing information over the genders (8·6); mean of age and age categories (2·1), school classes (10·5), school level (5·1) and secondary school level (50). Primary school includes the first 6 years of schooling.

	Level	Before (Control)	After (Intervention)	Missing (%)
**Number of participants**		1270	1264	0·47
**Gender: N (%)**	Male	609 (50·7)	542 (48·6)	8·6
	Female	592 (49·3)	574 (51·4)	
**Age: Mean (SD)**		13·43 (1·15)	13·38 (1·14)	2·1
**Age category: N (%)**	10–12	249 (19·9)	248 (20·1)	2·1
	13–14	829 (66·4)	827 (67·1)	
	15–18	171 (13·7)	158 (12·8)	
**School class number: N (%)**	5	26 (2·3)	24 (2·2)	10·5
	6	215 (18·7)	208 (18·6)	
	7	346 (30)	373 (33·4)	
	8	515 (44·7)	469 (42)	
	9	50 (4·3)	42 (3·8)	
**School level: N (%)**	Primary	241 (19·7)	232 (19·6)	5·1
	Secondary	981 (80·3)	952 (80·4)	
**Secondary school level: N (%)**	A	335 (51·1)	312 (51·1)	50
	B	296 (45·1)	278 (45·6)	
	C	25 (3·8)	20 (3·3)	

*SD: standard deviation.

**Table 2:** Distribution of questionnaire items and overall score before and after educational session of the intervention. Details about questions can be found in the supplementary material. Percentage of missing data designates the missing answers. Statistical tests comparing answers before and after educational session of the intervention included tests apart from McNemar test that was used for question number one regarding lung damage by smoking are too conservative because they do not consider correlation of answers from the same schoolchildren. For McNemar test intermediate correlation of pairs was assumed. P-values of suitable statistical tests comparing the two groups are adjusted for multiple comparisons by Benjamini-Hochberg method.

	Answers	Before (Control)	After (Intervention)	Missing (%)	Test type	Test statistic	p-value
**Number of participants**		1270	1264	0·47			
**Questions**							
**Q1: What part of the body (organs) are damaged by smoking?** Mean (SD*)		3·16 (1·32)	5·98 (1·39)	0·5	Two-sample *t*-test	52	< 0·0001
Lung (yes is correct): N (%)	No	15 (1·2)	4 (0·3)	0·5	McNemar’s	7	0·03
	Yes	1249 (98·8)	1253 (99·7)				
Heart (yes is correct): N (%)	No	487 (38·6)	104 (8·3)	0·6	Chi-squared	320	< 0·0001
	Yes	776 (61·4)	1153 (91·7)				
Eyes (yes is correct): N (%)	No	1181 (93·5)	402 (32)	0·6	Chi-squared	1018	< 0·0001
	Yes	82 (6·5)	855 (68)				
Bones (yes is correct): N (%)	No	1102 (87·2)	248 (19·7)	0·5	Chi-squared	1150	< 0·0001
	Yes	162 (12·8)	1009 (80·3)				
Skin (yes is correct): N (%)	No	736 (58·2)	112 (8·9)	0·5	Chi-squared	684	< 0·0001
	Yes	528 (41·8)	1145 (91·1)				
Gums (yes is correct): N (%)	No	393 (31·1)	172 (13·7)	0·5	Chi-squared	109	< 0·0001
	Yes	871 (68·9)	1085 (86·3)				
Vessels (yes is correct): N (%)	No	933 (73·9)	233 (18·5)	0·6	Chi-squared	774	< 0·0001
	Yes	330 (26·1)	1024 (81·5)				
All 7 organs are damaged: N (%)	Incorrect answer	1236 (97·8)	579 (46·1)	0·5	Chi-squared	834	< 0·0001
	Correct answer	28 (2·2)	678 (53·9)				
**Q2: Do increased cigarette prices result in a reduction of smoking?**: N (%)	Incorrect answer	871 (75·9)	511 (41·2)	5·8	Chi-squared	294	< 0·0001
	Correct answer	276 (24·1)	730 (58·8)				
**Q3: Does smoking only 1**–**4 cigarettes per day over many years not carry significant health risks?**: N (%)	Incorrect answer	156 (13·4)	103 (8·2)	4·5	Chi-squared	16	0·0002
	Correct answer	1011 (86·6)	1150 (91·8)				
**Q4: Most smokers believe that they can quit smoking all by themselves. How many adults can quit smoking all by themselves?** (Few is correct answer): N (%)	Half	38 (3·1)	13 (1)	2·6	Chi-squared	356	< 0·0001
	Quarter	428 (35·1)	69 (5·5)				
	Few	753 (61·8)	1167 (93·4)				
**Q5: Is water pipe/hookah/shisha‘s smoking not as dangerous as cigarette smoking?**: N (%)	Incorrect answer	400 (36·2)	116 (9·3)	7·1	Chi-squared	246	< 0·0001
	Correct answer	706 (63·8)	1133 (90·7)				
**Overall score (15 maximal): Mean (SD)**		5·95 (3·06)	12.12 (2·60)	0·5	Two-sample *t*-test	55	< 0·0001

*SD: standard deviation.

All p-values in Table 2 with the exception one question regarding lung damage by smoke are very low and we can conclude that there is very strong evidence that the children increased their knowledge about smoking effects as a result of the intervention. In terms of improving knowledge about lung damage by smoke, only moderate evidence was found for a learning effect. This is mainly because most of the children already knew that smoking has negative effects on the lungs before the educational session.

### 3.2 Analysis of subgroups of children followed by feedback

Knowledge about the effects of smoking increased at about the same rate for both genders. The percentage of questions answered correctly increased at about the same rate for all subgroups of children after the educational session (Table 3). In addition, the estimated increase in scores from before to after for most subgroups of children was between 6 and 7, roughly a doubling of scores, from about 40% of scores to about 80% of scores. Only for children over 15 and in secondary school level B did the increase in knowledge tend to be smaller (4–8 points and 5–8 points, respectively), but the differences are within standard deviations. The higher increase in total scores for children in secondary school level C (7–6) was also within standard deviations. In all subgroups of children, there was good evidence of a learning effect of the teaching, as the statistical tests for the change in total score yielded low p-values (p < 0·0001).

**Table 3:** Mean and standard deviation of overall score for different subgroups (gender, age category groups, school levels and secondary school levels) of schoolchildren participating in the intervention. Mean difference between before and after educational session score were calculated with standard deviation and the SMD (standardized mean difference (Hedge’s g)) with 95% CI. The overall score was compared with the formal *t*-test also in subgroups of children. Results of statistical tests comparing answers before and after educational event. P-values adjusted for multiple comparisons by Benjamini-Hochberg method are shown.

	Level	Before (Control)	After (Intervention)	Difference (mean)	SMD with 95% CI	Test type	Test statistic	p-value
**Number of participants**		1264	1258					
**Gender:** **Mean (SD)**	Male	6·07 (3)	12·15 (2·57)	6·08 (2·81)	2·17 [2·02; 2·31]	Two-sample *t*-test	37	< 0.0001
	Female	5·72 (3·07)	12·14 (2·63)	6·42 (2·86)	2·24 [2·1; 2·39]	Two-sample *t*-test	38	< 0.0001
**Age category** **Mean (SD)**	(Tobler et al., 2000, Wrotgte, 2019)	5·58 (3·02)	12·36 (2·49)	6·78 (2·77)	2·45 [2·21; 2·68]	Two-sample *t*-test	27	< 0.0001
	(McAfee et al., 2013, Xiao et al., 2013)	5·86 (3·01)	12·17 (2·6)	6·31 (2·81)	2·24 [2·12; 2·37]	Two-sample *t*-test	46	< 0.0001
	(Bala et al., 2017, von Elm et al., 2007)	6·66 (3·08)	11·49 (2·61)	4·83 (2·87)	1·68 [1·43; 1·93]	Two-sample *t*-test	15	< 0.0001
**School level** **Mean (SD)**	Primary	5·42 (3·06)	12·29 (2·58)	6·87 (2·84)	2·42 [2·18; 2·66]	Two-sample *t*-test	26	< 0.0001
	Secondary	6·05 (3·03)	12·13 (2·59)	6·08 (2·82)	2·16 [2·04; 2·27]	Two-sample *t*-test	47	< 0.0001
**Secondary school level:** **Mean (SD)**	A	6·38 (3·09)	13·02 (2·18)	6·64 (2·69)	2·47 [2·26; 2·67]	Two-sample *t*-test	32	< 0.0001
	B	5·97 (3·03)	11·81 (2·53)	5·84 (2·8)	2·08 [1·88; 2·29]	Two-sample *t*-test	25	< 0.0001
	C	4·8 (2·14)	12·4 (2·35)	7·6 (2·27)	3·34 [2·42; 4·26]	Two-sample *t*-test	11	< 0.0001

SD: standard deviation, CI: confidence interval.

During the prevention intervention, the children took the opportunity to discuss additional questions with the pulmonologist and the thoracic surgeon (supplementary material).

Following the first intervention sessions, we received the feedback from the teachers that some scholars spoke to unknown adults waiting at the tramway station about their smoking habits and suggested an immediate smoking cessation due to the potential of adverse events that may result from continued smoking. In other situations, scholars demanded from their parents to stop immediately for similar reasons. This controversial behavior of addressing persons with the request for smoking cessation was discussed with the teachers and the intervention from then on included a clear statement that the intervention is intended to influence the scholars knowledge level and understanding, and that smoking is an addictive disease that usually cannot be easily terminated by simply requesting a cessation. This added explanation during the intervention successfully prevented further unsolicited smoking cessation requests.

All children had the opportunity to give feedback, concerning the whole intervention in general, within a few days or weeks after the intervention: 443 children provided such a feedback. The feedback questionnaire included structured questions with predefined answers as well as open questions, where open questions and answers were requested. These are shown in Table A1 (supplementary material). The distribution of the schoolchildren characteristics and answers in the feedback forms of the intervention are provided in Table 4, Table 5.

**Table 4:** Distribution of characteristics of schoolchildren who handed in the feedback questionnaire after the intervention. Percentages of missing data are shown in the last column. Primary school includes the first 6 years of schooling. SD: standard deviation.

	Level	Overall	Missing (%)
**Number of participants**		443	
**Gender: N (%)**	Male	183 (43·5)	5
	Female	238 (56·5)	
**Age (mean (SD))**		13·17 (1·17)	2·7
**Age category: N (%)**	10–12	128 (29·7)	2·7
	13–14	245 (56·8)	
	15–18	58 (13·5)	
**School class number: N (%)**	5	1 (0·3)	17·8
	6	114 (31·3)	
	7	129 (35·4)	
	8	97 (26·6)	
	9	23 (6·3)	
**School level: N (%)**	Primary	115 (28·2)	7·9
	Secondary	293 (71·8)	
**Secondary school level: N (%)**	A	80 (61·1)	70·4
	B	51 (38·9)	
	C	0 (0·0)

**Table 5:** Feedback questionnaire handed in by 443 schoolchildren after the intervention. Answers are summarized in counts and percentages. Percentage of missing data are shown in the last column.

	Answers	OverallN (%)	Missing (%)
**Number of participants**		443	
**Question regarding presented pictures (possible answers)**			
Interesting	Yes	392 (97·5)	9·3
	No	10 (2·5)	
Scary	Yes	181 (52·5)	22·1
	No	164 (47.5)	
Informative	Yes	364 (96)	14·4
	No	15 (4)	
**Question regarding direct talk with the patient (possible answers)**			
Interesting	A lot	353 (81·7)	2·5
	A little	68 (15·7)	
	Not at all	11 (2·5)	
Duration	Spot on	344 (79·1)	1·8
	Too short	33 (7·6)	
	Too long	58 (13·3)	
**Question regarding participation in a pulmonary function test (possible answers)**			
Interesting	A lot	274 (63·9)	3·2
	A little	137 (31·9)	
	Not at all	18 (4·2)	
**General evaluation of the educational session in hospital (possible answers)**			
Participant liked this educational session	A lot	328 (75·8)	2·3
	Moderately	97 (22·4)	
	A little	8 (1·8)	
Participant found this educational session as informative	A lot	342 (80·9)	4·5
	Moderately	74 (17·5)	
	A little	7 (1·7)	

Most children stated that the pictures and films were interesting and informative. The patient interview was mostly rated as interesting and of appropriate length. Nearly-two third of the scholars rated the lung function test as very interesting.

## 4 Discussion

In this smoking prevention project at the university hospital Zurich, we showed that a school-class-based intervention by thoracic surgeons and pulmonologists is feasible and increases the knowledge on the topic by doubling the percentage of correctly answered questions provided by the scholars.

Similar interventions including patients with tobacco-related diseases have been evaluated by physicians and medical students from the Education Against Tobacco network in randomized controlled trials with follow-up data showing effectiveness of the intervention in reducing smoking onset or increasing smoking cessation rates (Lisboa et al., 2019, Brinker et al., 2015). These two important studies have confirmed that the complex set of conditions may yield favorable results likely because of the hospital setting, the content of the intervention, and the presence of a health professional as conductor of the intervention.

Our as information-only classified hospital-based intervention program with subsequent strong interactive component from session to session with individualized additional questions successfully increased 10–16-year-old scholars’ knowledge on selected smoking related questions and improved the effectiveness of smoking prevention. We have shown that it is feasible to conduct such an intervention in a university hospital setting resulting in positive acceptance of the intervention by scholars and teaching staff. Whether this intervention reduces uptake of smoking in the years following the intervention was not investigated and therefore remains unknown. Similar hospital-based programs or school-based interventions provided by health educators or physicians do exist but have rarely been studied or evaluated systematically. Positive effects of a hospital-based intervention were reported by Schoenfeld and colleagues (Stamm-Balderjahn et al., 2012), where significantly fewer scholars in the intervention group than in the control group began smoking after the intervention (p < 0·001). The rate of remaining a non-smoker were four times higher in the intervention group (95% CI: 1·66-10·36) and interestingly girls benefited from the intervention more than boys. Furthermore, 16% of smokers in the intervention group and 17·6% in the control group quitted smoking (Stamm-Balderjahn et al., 2012).

In early investigations Coe and colleagues (Coe et al., 1982) smoking prevention program organized by first-year medical students for adolescents in middle schools was focused on resisting to smoke and understanding the intentions of cigarette commercials. The authors suggest that implementation of smoking prevention programs in school-settings may be effective in reducing the prevalence of cigarette smoking (Coe et al., 1982). In 2006 Chou and colleagues (Chou et al., 2006) published randomized intervention for adolescents in China with 1 year follow-up implemented by US-trained health educators from the Wuhan Center for Disease Control and Prevention. This smoking prevention curriculum did not demonstrate a primary prevention effect but showed potential for secondary prevention for scholars aged 12·5 years. At the 1-year follow-up, smoking had increased more rapidly in the control schools than in the program schools. This program prevented progression to smoking among boys who were baseline ever smokers (95% CI: 0·23-0·88) (Chou et al., 2006). Thomas and colleagues (Thomas et al., 2013) presented a comprehensive review of 49 randomized controlled trials of school-based interventions and showed a 12% reduction in smoking initiation compared to the controls at longest follow-up time-point. The social competence and social influences interventions prevented children and adolescents from beginning to smoke (95% CI: 0·30-0·88) at one year and at the longest follow-up.

The multimodal- and on information only based interventions were often judged as ineffective (Thomas et al., 2013). Interestingly, Brinker et al. (Brinker et al., 2016) developed a photoaging app to reduce smoking prevalence in secondary schools. This group evaluated its effectiveness regarding smoking prevalence and attitudes towards smoking in a RCT with pupils aged 12 years. The measurements are performed at baseline and at 6, 12 and 24 months post intervention via a questionnaire with a random cotinine saliva measurement at 24 months (Brinker et al., 2016). This study will be able to show the difference of change in smoking prevalence in the intervention group vs the control group at 24 months (primary outcome). Furthermore, changes in smoking-related attitudes, the number of new smokers and quitters and the change in the number of never-smokers between the two groups (secondary outcomes) are additional interesting outcomes (Brinker et al., 2016). Such a RCT by Lisboa et al. showed that the intervention encourages quitting and prevents smoking onset particularly in male scholars aged 12–21 (Lisboa et al., 2019). This provides some support for the effectiveness of community interventions in helping to decrease the initiation of smoking and promotes smoking cessation in young people (Lisboa et al., 2019, Sowden et al., 2003, Carson et al., 2011, Carson et al., 2017).

Since the smoking habits in adolescents are changing, the research group from Moeller and colleagues (Mozun et al., 2020) found in a recent study, that Swiss schoolchildren often combined smoking cigarettes with shisha-smoking and electronic cigarette use and was associated with more respiratory related symptoms than in never smokers. They recommended smoking prevention strategies that include all forms of smoking (Mozun et al., 2020).

One specific aspect needs to be mentioned: We wanted the scholars to see this intervention as information for themselves to reduced their susceptibility to tobacco addiction and wanted to prevent them from ‘missionizing’ parents, relatives, or friends, mentioning that smoking is an individual decision and that it is not their duty to convince others to quit smoking.

The visit of the scholars in the hospital, including contact with patients and physicians, presented films and pictures, possibility of lung function testing, combined with an educational session was found to be excellent by more than two thirds of the children and was rated as very informative by more than 80% of the children (Table 4, Table 5).

In the context of COVID-19, several researchers have pointed out the link between the infection and adverse outcomes in smokers. Recently, Patanavanich and colleagues pointed out that smoking is a risk factor for the progression of COVID-19 compared to never smokers (Patanavanich and Glantz, 2020) and Adams and colleagues detected a lower medical vulnerability to severe COVID-19 among young adult non-smokers (Adams et al., 2020). In the current difficult times, it may be a chance of the pandemic to encourage and support quitting tobacco use in adolescents by offering school-based interventions in a health-care setting.

## 5 Conclusions

We presented here a hospital-based intervention program in schoolchildren aged 10–16 to prevent smoking initiation or promote early cessation. The program was well received and showed a significant increase in knowledge as assessed by a questionnaire. Furthermore, this program is feasible in a university hospital setting and may possibly be transferrable to similar settings. Whether this intervention has a significant impact on smoking initiation remains to be determined in further research.

## 6 Strengths

The hospital-based intervention program is feasible and confirms very clear improvements of knowledge about harmful impacts of smoking in all subgroups.

## 7 Limitations of this study

We collected the responses only immediately after the educational session. The approach and intensity of the school class activities in addition to the educational session was at the discretion of the teachers and the effect on smoking uptake was not evaluated.

It was not tested how long the acquired knowledge is retained and if the improved knowledge leads to reduced smoking initiation.

It remains to be determined in future studies whether there might be a long-term preventive effect. As the questionnaires were submitted anonymously, they could not be paired for analysis and special statistical methods had to be used.

## Funding

Department of Thoracic Surgery, University Hospital Zurich.

## Ethical statement

BASEC-Nr. Req-2022-00035.

## Declaration of Competing Interest

The authors declare that they have no known competing financial interests or personal relationships that could have appeared to influence the work reported in this paper.

## Data Availability

Data will be made available on request.
