# Supplementary material for: Smoking prevention intervention with school classes in university hospital by thoracic surgeon und pulmonologist. The Zurich prevention project
Source: Prev Med Rep. 2022 Aug 30;29:101964. doi: 10.1016/j.pmedr.2022.101964 (PMC9502329; doi:10.1016/j.pmedr.2022.101964)
Supplement: Supplementary data 1 [file mmc1.docx]

**Appendices**

**Supplementary Material**

**A1. Intervention provided by the pulmonologist:**

The 45-75 minutes contact with the pulmonologist starts with a short slide presentation of the function of the lung, how lung function tests are performed and what effects of smoking on different organ systems occur. Hookah, or waterpipe smoking (also called nargile or shisha) was covered, including a brief explanation of functioning of the shisha device, comparative amounts of inhaled components, risk of infection transmission, etc.) and illustration of effects of smoking on vasculature. Briefly, some smoking cessation strategies are mentioned, and all topics mentioned in the questionnaire are included without explicitly mentioning the questionnaire or the same wording. One or two volunteers were chosen from the audience for a lung function test and an exhaled carbon monoxide (eCO) measurement were performed. The largest part of the intervention (20-30 minutes) with the pulmonologist was used for an interview with a patient, who received a lung transplant due to advanced COPD (chronic obstructive pulmonary disease) with or without lung emphysema. The patient was present in the auditorium and scholars had the opportunity to ask the questions at any time, directed to the physician or the patient. The questions addressed the patient's experience of starting to smoke and the increase in use over time, addiction, and adverse effects perception, attempts to stop smoking and final smoking cessation, which ultimately made a lung transplant possible. Amount of medication required as a transplant recipient and quality of life before and after transplantation were also intensively discussed. At the end of the interview session the patient was asked about their own learned lessons with smoking and the smoking cessation process and if they wanted to ad any recommendation for the scholars. The same patient was interviewed twice for both school classes. In our experience the patients enjoyed these interviews and usually volunteered for further intervention sessions for scholars. Many different patients have been interviewed so far in the decade of interventions for schools.

**A2. Intervention provided by the thoracic surgeon:**

A brief, age-appropriate introduction of the anatomy and physiology of the lungs and the breathing process was given, highlighting in particularly the vulnerability of all organs protected by the chest cavity. For this overview, we used not only a slide presentation but also three-dimensional homologous anatomical models, models that represent anatomical structures in life-size or magnified form (used in the training of students and doctors) to better illustrate the topographical anatomy. In the following, the facts on the morbidity and mortality of smokers as well as on the toxic ingredients of cigarette smoke and its harm to various organs were presented. In this part we focussed as well on examples of well-known personalities from sports and other professions who either never smoked or succeeded in quitting smoking (positive examples of non-smokers). The children were educated that smoking is not compatible with an active, sporty lifestyle in the long run, that it should not be used to stay slim, and that even a small number of cigarettes can lead to addiction and harm.

One of the key messages of this section, was that it is much more difficult to quit smoking than to be able to refrain from smoking as a young person. Subsequently, two video sequences of a minimal invasive operation were shown, presenting the lungs of a non-smoker and, in the second sequence, the lungs of a heavy smoker with emphysema and lung cancer resected with the surgical instruments. These sequences were explained in detail by the thoracic surgeon and the subsequent questions of the school children concerning the slide show and the video were answered. At the conclusion of both parts of the intervention, we provided age-appropriate internet links with general information on the topic of addiction prevention and motivated the children to commit as individuals or even better as a group not to start smoking for a certain period of time (e.g. one year) in order to win prizes for the group (travel vouchers or similar).

We further emphasized that this intervention was intended to influence their individual live and development and requested them to be tolerant with adults (their parents) who may be smokers and have similar difficulties to stop smoking as they heared of in the current intervention session. This comment was made to prevent the young people from reacting strongly to adults smoking shortly after the sessions (as often reported by the teachers).

**A3. School system in Canton of Zurich**

All children go for to 2 years to the Kindergarten (preschool), followed by 6 years of primary school and 3 years of secondary school or alternatively 6 years of cantonal school (Gymnasium). In primary school, all children are taught in mixed classes at all levels. In secondary school, pupils are separated in several sections (level: A, B, C) according to their capacities. Pupils with the highest capacities and those who aspire for an academic career go to cantonal school (Gymnasium). During the sixth year of primary school, children are normally 12-13 years old, at the end of secondary school about 16 years.

**Additional open questions and answers**

| Questions | Answers (abbreviated) |
| --- | --- |
| Is passive smoking also harmful? | Yes, but not as much as active smoking |
| Is cannabis / joint smoking also harmful? | Yes, one joint equals about 8 cigarettes in terms of damage to the lungs. |
| Is an electronic cigarette also harmful? | Possibly yes, but likely less damaging than cigarette smoking, but long-term effects are largely unknown. |
| Does one also get addicted to “Niele-smoking”? (implicated smoking of dried Clematis vitalba)? Is it harmful? | No nicotine involved, therefore not same potential for addiction, combustion process similar, so some harm likely since inhaling smoke. |
| Can parents donate lungs or parts of lungs for their children? | Yes, but not done in Switzerland. Japan has done this a lot in the past. |

Table A1: Frequent additional open questions to pulmonologist and thoracic surgeon and abbreviated answers.

**Supplementary statistical analysis**

To consider correlation of paired observations the following methods were used:

- Numerical answers and the overall score were analyzed with a 1-sample t-test of the changes, while accounting for correlation in the derivation of a pooled standard deviation ($sd_{pooled}=\sqrt{sd_{1}+sd_{2}-2\times(cor\times sd_{1}\times sd_{2})}$)
- Categorical answers were analyzed with the McNemar’s test, by analyzing the two most extreme contingency tables of paired data that were constructed by only knowing the margins.

As a first approximation the testing methods for uncorrelated data (chi-square test or fisher’s exact test for categorical data, two-sample t-test for numerical data) were used. The methods for uncorrelated data give slightly more conservative results, i.e., higher p-values. Whenever the testing methods for uncorrelated data gave very clear results (p-value <0·0001) then the more complicated statistical tests mentioned above were not applied, because we could be sure that the resulting p-value would also be below 0·0001. The resulting p-values were corrected for multiple testing by the Benjamini-Hochberg method.
